# Strangulated ileus due to anterior enterocele following robot-assisted radical cystectomy: A case report of rare complication managed with minimally invasive surgery^1^

**DOI:** 10.1016/j.ijscr.2025.111839

**Published:** 2025-08-18

**Authors:** Seiya Yamamoto, Yoshiaki Fujii, Masaaki Kurimoto, Hiroki Takahashi, Hirozumi Sawai, Shuji Takiguchi

**Affiliations:** a Department of Gastroenterological Surgery, Nagoya City University Graduate School of Medical Sciences, Nagoya, Aichi 467-8601, Japan; b Narita Memorial Hospital, Toyohashi, Aichi 441-8029, Japan

**Keywords:** Anterior enterocele, Case report, Intracorporeal anastomosis, Strangulated ileus

## Abstract

**Introduction:**

The incidence of anterior enterocele following robotic radical cystectomy is reported to be approximately 3 %, making it a rare complication. We experienced a case in which a patient developed an anterior enterocele and strangulated ileus through the urethral excision site, for which intracorporeal anastomosis proved to be an effective treatment under a specific and limited clinical situation.

**Presentation of case:**

A 72-year-old woman underwent a robot- assisted radical cystectomy for bladder cancer. At nine months post-operation, she presented with small bowel evisceration resulting from dehiscence of an anterior enterocele accompanied by strangulated ileus. Laparoscopic small bowel resection with intracorporeal anastomosis (IA) was performed. Oral intake of food was resumed on post-operative day 3, and the patient was discharged without complications on post-operative day 10.

**Discussion:**

In cases of strangulated ileus with dilated bowel loops, minimally invasive surgery incorporating IA can be safely performed by temporarily utilizing an intestinal bulldog clamp for the bowel, employing indocyanine green fluorescence imaging, and selecting an appropriate anastomotic technique. If an adequate surgical environment is maintained, IA may be a viable option for the treatment of strangulated ileus.

**Conclusion:**

IA may be a viable option for treating strangulated ileus in a limited situation.

## Highlights

• We report a rare case of anterior enterocele via urethral excision site following robot-assisted radical cystectomy.
• Laparoscopic hernia reduction and IA performed using ICG imaging and temporary intestinal clamp.
• Minimally invasive approach demonstrated safe and effective outcomes with early oral intake and discharge.

## 1 Introduction

In radical cystectomy involving anterior vaginal wall resection and urethrectomy, the periurethral fascia and ligamentous supporting structures attached to the pubic symphysis are resected [1]. Several reports have described herniation involving this weak area as well as pelvic organ prolapse caused by wound dehiscence. Among these, anterior enterocele following robotic radical cystectomy has been reported to occur in approximately 3 % of cases, indicating that it is a rare but notable complication [2]. To date, no cases of intracorporeal anastomosis for strangulated bowel obstruction have been reported. We treated a patient who developed an anterior enterocele and strangulated ileus through the urethral excision site, for which intracorporeal anastomosis (IA) proved to be an effective treatment. This case was reported in accordance with the SCARE criteria [3].

## 2 Presentation of case

The patient was a 72-year-old woman who had previously undergone robot-assisted left nephroureterectomy and total cystectomy at the age of 71 for left renal pelvic and bladder cancer. Although her early postoperative course was uneventful, she experienced early recurrence with pulmonary metastases and left renal hilar lymphadenopathy, for which she subsequently received chemotherapy.

Approximately 8 months post-operation, small bowel prolapse through the urethral excision site was observed during an outpatient visit. It was possible to manually reduce the bowel; therefore, the patient was managed conservatively with observation.

**Fig. 1:**
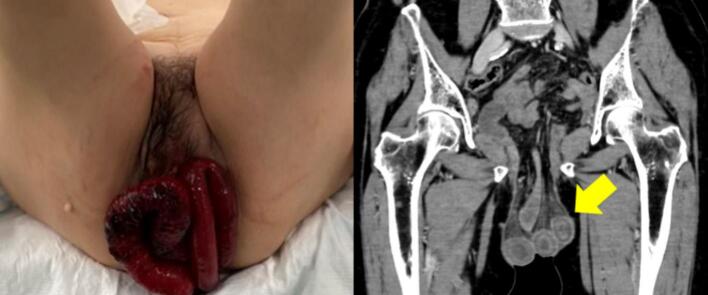
Physical examination: Evisceration of dark red small intestine was observed from the urethral extirpation site. The abdominal contrast-enhanced computed tomography scan findings: the arrow indicates thickened ileum wall and oedematous mesentery.

**Fig. 2:**
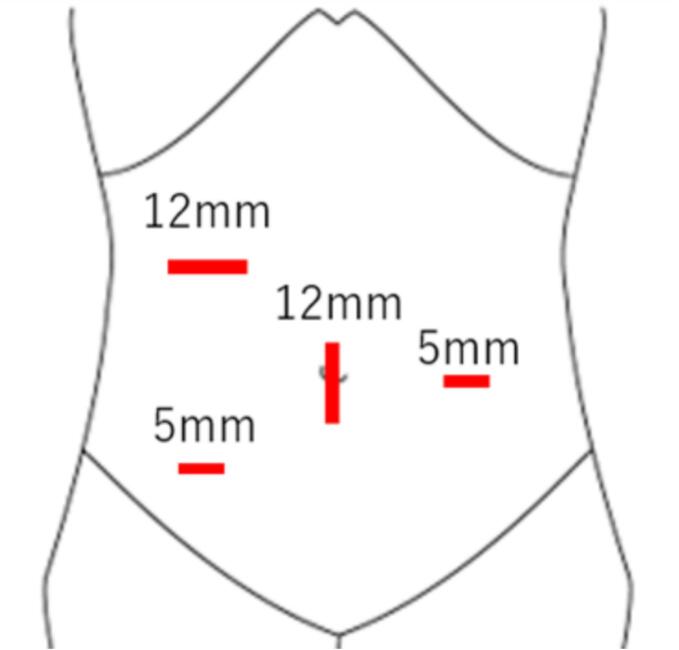
Port settings; 12-mm trocar inserted at the umbilicus, and 12 mm in the right upper abdomen and 5 mm ports bilaterally in the flanks.

**Fig. 3:**
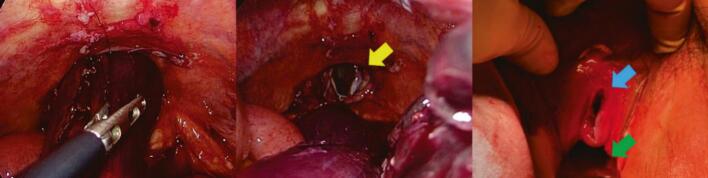
Small intestine had prolapsed through the urethral excision site and approximately 1 m was incarcerated and necrotic. After laparoscopic hernia reduction. The yellow arrow indicates urethral extraction site. The blue arrow indicates the urethral extraction site. The green arrow indicates vagina.

**Fig. 4:**
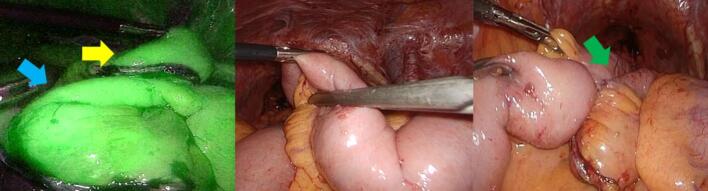
Indocyanine green fluorescence imaging: The yellow arrow indicates the oral bowel stump, and the blue arrow indicates the anal bowel stump. A temporary intestinal bulldog clamp was applied to the oral side of the bowel intended for anastomosis. The entire surgical procedure was completed. The anastomotic site is indicated by the green arrow.

Nine months after surgery, the patient presented to our hospital with lower abdominal pain. Physical examination revealed massive small bowel prolapse through the urethral excision site, with signs of oedema and ischemic changes. Contrast-enhanced abdominal computed tomography (CT) revealed ileal wall thickening and mesenteric oedema (Fig. 1). Emergency laparoscopic surgery was performed using a 12-mm trocar inserted into the umbilicus and the right upper abdomen, and 5-mm ports were inserted bilaterally into the flanks (Fig. 2). Intraoperatively, the small intestine had prolapsed through the urethral excision site, and approximately 1 m of it was incarcerated and necrotic. Owing to strong incarceration, reduction via laparoscopy would have been difficult. Therefore, perineal resection of the necrotic bowel was performed using linear staplers to reduce the volume of the prolapsed bowel. Subsequently, laparoscopic reduction of the hernia was performed through the urethral excision site (Fig. 3). Perineal closure was performed, and the remaining necrotic small bowel tissue was resected within the abdominal cavity. The resected bowel was extracted through an umbilical incision. Before IA was performed, indocyanine green (ICG) fluorescence imaging was used to confirm adequate blood flow to the bowel stump. To prevent the spillage of intestinal contents during anastomosis, the oral side of the bowel was clamped using a temporary intestinal bulldog clamp. Approximately 15 cm of the terminal ileum from the ileocecal region remained and overlap anastomosis (OLA) was performed using a linear stapler. The enterotomy was closed using a single-layer full-thickness suture with barbed suture material, the mesenteric defect was closed, and the procedure was concluded (Fig. 4) (Supporting Video).

The operation duration was 201 min and the estimated blood loss was 30 mL. Oral intake resumed on postoperative day 3, and the patient was discharged without complications on postoperative day 10. No postoperative recurrence was observed; however, the patient died approximately 10 months later following progression of primary bladder cancer.

## 3 Discussion

Radical cystectomy with anterior vaginectomy and urethrectomy disrupts periurethral fascia and the ligamentous support of the pubic symphysis, increasing the risk of pelvic organ prolapse (POP) in female patients [4]. Previous reports described anterior enterocele after radical cystectomy typically involving bowel evisceration through a vaginal defect [5]. However, in this case, the bowel prolapse occurred at the urethral extraction site. A narrower urethral defect may increase the risk of strangulation owing to the limited space for the prolapsed bowel.

At 8 months postoperatively, the patient presented with a reducible anterior enterocele through the urethral excision site. Given the absence of symptoms such as pain, bowel obstruction, or infection at that time, conservative management was decided upon, influenced by the patient's history of advanced bladder cancer and recent radical pelvic surgery, which made us initially hesitant to pursue elective reoperation, considering the overall physical burden. However, in retrospect, early elective repair might have prevented the subsequent development of strangulation, bowel necrosis, and the need for emergency resection. In such cases, even if initially asymptomatic, prompt surgical intervention may be warranted to reduce the risk of life-threatening complications. An anterior enterocele might be underdiagnosed in patients with progressive disease or limited life expectancy following a cancer diagnosis, as clinical focus tends to remain on oncologic control. In this case, the lack of surgical intervention for the anterior enterocele due to cancer history is a point of reflection, emphasizing the importance of proactive assessment and intervention even in patients with advanced-stage malignancy.

In the present case, a large volume of necrotic bowel prolapsed through the urethral site. As the anal-side transection was located approximately 15 cm from the ileocecal valve, extracorporeal anastomosis (EA) would have required extensive mobilization of the cecum and ascending colon, which may have necessitated a larger incision for anastomosis. Therefore, we opted for IA using OLA, because of its effectiveness in such scenarios [6]. To date, there have been no reports of IA being performed for strangulated ileus. This may be because of the limited intra-abdominal working space caused by dilated bowel loops, which makes securing an adequate surgical field difficult, as well as the increased risk of intra-abdominal contamination and infection from the leakage of bowel contents from the distended intestine. In this case, following the complete extracorporeal removal of the necrotic bowel, the strategic use of an intestinal clamp to occlude the oral bowel segment prevented intraperitoneal contamination and allowed for safe anastomosis. All procedures were performed by a surgeon certified under the Endoscopic Surgical Skill Qualification System (ESSQS) of the Japan Society for Endoscopic Surgery, who had performed over 50 intracorporeal anastomoses for colorectal cancer. We acknowledge the importance of ensuring sufficient distance between the anastomosis and the ileocecal valve to maintain a safe pressure gradient and minimize the risk of postoperative complications.

Furthermore, ICG fluorescence imaging plays a critical role in confirming adequate perfusion prior to anastomosis. ICG imaging has been widely used to assess anastomotic blood flow and is effective in evaluating bowel viability in cases of strangulated hernia or ischemia [7]. In this case, it supported the decision to proceed with IA.

Previous reports have described performing closure of the enterotomy in OLA using a linear staple [8]. In this case, hand-sewn full-thickness closure using barbed sutures was performed, because closing the enterotomy with a linear stapler in small intestine anastomosis may result in partial resection of the bowel wall at the outlet because of the narrower diameter of the small intestine compared with that of the colon, thereby increasing the risk of obstruction. No post-operative anastomotic stenosis was observed.

IA offers several advantages over EA, including requiring a smaller incision size, a lower incidence of postoperative ileus, and resulting in shorter post-operative hospital stays [9]. However, some reports have indicated an increased risk of surgical site infections (SSI) with IA [10]. In the present case, no SSI or intra-abdominal abscess was observed, and early resumption of oral intake and discharge was achieved. Moreover, in cases where the anastomotic site is located close to the ileocecal region, IA allows for minimal bowel mobilization and minimal incision length, demonstrating its effectiveness as a minimally invasive surgical option.

To our knowledge, this is the first reported case in which IA was performed with the presence of a strangulated ileus. Thus, IA may be a feasible option when appropriately utilizing ICG fluorescence imaging and intestinal clamps to secure an adequate surgical field. This minimally invasive surgical approach may be valuable for treating strangulated ileus.

## 4 Conclusion

Intracorporeal anastomosis (IA) may be a feasible and minimally invasive option for the treatment of strangulated ileus, particularly when using adjuncts, such as ICG imaging and temporary intestinal clamping.

The following is the supplementary data related to this article.

Supporting Video Laparoscopic resection and intracorporeal anastomosis for strangulated ileus caused by small bowel prolapse through the urethral excision site. Key steps include hernia reduction, indocyanine green-based perfusion assessment, and side-to-side overlap anastomosis with barbed suture closure. Supporting Video

## Informed consent

This study was conducted according to the principles of the Declaration of Helsinki. Written informed consent was obtained from the patient for publication of this case report and accompanying images. A copy of the written consent is available for review by the Editor-in-Chief of this journal on request.

## Ethical approval

Ethical approval was not required for this single-patient case report in accordance with the policy of our institution.

## Ethical statement

Ethical approval was not applicable to this case report in accordance with the policy of our institutional review board.

## Author contributions

Seiya Yamamoto contributed to the conception and design of the study, manuscript drafting, and overall structure development.

Yoshiaki Fujii supervised the project, revised the manuscript critically for important intellectual content, and contributed to the narrative and structural organization.

Masaaki Kurimoto participated in the clinical management of the patient and assisted in data collection.

Hirozumi Sawai and Hiroki Takahashi contributed to manuscript organization and structural refinement.

Shuji Takiguchi provided overall supervision of the study and approved the final manuscript.

## Authorship declaration

All authors have made substantial contributions to the manuscript in accordance with the ICMJE criteria for authorship.

## Funding

This research did not receive any specific grant from funding agencies in the public, commercial, or not-for-profit sectors.

## Funding

The authors declare that no funding was received by any of the authors for the research, authorship, or publication of this article.

## Declaration of competing interest

The authors declare no conflicts of interest.
